# Supplementary material for: Changes in the Proteome in the Development of Chronic Human Papillomavirus Infection—A Prospective Study in HIV Positive and HIV Negative Rwandan Women
Source: Cancers (Basel). 2021 Nov 28;13(23):5983. doi: 10.3390/cancers13235983 (PMC8656715; doi:10.3390/cancers13235983)
Supplement: Supplementary file 1 [file cancers-13-05983-s001.zip › cancers-1472823-SI.pdf]

Article

Changes in the Proteome in the Development of Chronic Human Papillomavirus Infection—A Prospective Study in HIV Positive and HIV Negative Rwandan Women

Emile Bienvenu [1], Marie Francoise Mukanyangezi [1], Stephen Rulisa [2], Anna Martner [3], Bengt Hasséus [4], Egor Vorontsov [5], Gunnar Tobin [6] and Daniel Giglio [6,7,*]

[1] College of Medicine and Health Sciences, University of Rwanda, KK 737 Street, Gikondo, Kigali, Rwanda; ebienvenu3@gmail.com (E.B.); francizi@yahoo.fr (M.F.M.)
[2] College of Medicine and Health Sciences, University of Rwanda, University Teaching Hospital of Kigali (UTHK), KN 4 Avenue, Kigali, Rwanda; s.rulisa@gmail.com
[3] TIMM Laboratory, Sahlgrenska Center for Cancer Research, Department of Infectious Diseases, Institute of Biomedicine, Sahlgrenska Academy, University of Gothenburg, 41390 Gothenburg, Sweden; anna.martner@microbio.gu.se
[4] Department of Oral Medicine and Pathology, Institute of Odontology, Sahlgrenska Academy, University of Gothenburg, 40530 Gothenburg, Sweden; bengt.hasseus@odontologi.gu.se
[5] Proteomics Core Facility, Sahlgrenska Academy, University of Gothenburg, 40530 Gothenburg, Sweden; egor.vorontsov@gu.se
[6] Department of Pharmacology, Sahlgrenska Academy, University of Gothenburg, 40530 Gothenburg, Sweden; gunnar.tobin@pharm.gu.se
[7] Department of Oncology, Sahlgrenska Academy, University of Gothenburg, Sahlgrenska University Hospital, 41345 Gothenburg, Sweden
* Correspondence: daniel.giglio@pharm.gu.se

Citation: Bienvenu, E.; Mukanyangezi, M.F.; Rulisa, S.; Martner, A.; Hasséus, B.; Vorontsov, E.; Tobin, G.; Giglio, D. Changes in the Proteome in the Development of Chronic Human Papillomavirus Infection—A Prospective Study in HIV Positive and HIV Negative Rwandan Women. *Cancers* **2021**, *13*, 5983. https://doi.org/10.3390/cancers13235983

Academic Editor: Brian Gabrielli

Received: 4 November 2021
Accepted: 25 November 2021
Published: 28 November 2021

Publisher's Note: MDPI stays neutral with regard to jurisdictional claims in published maps and institutional affiliations.

Copyright: © 2021 by the authors. Licensee MDPI, Basel, Switzerland. This article is an open access article distributed under the terms and conditions of the Creative Commons Attribution (CC BY) license (https://creativecommons.org/licenses/by/4.0/).

Supplementary methods

HPV screening and cytology

The HPV screening test was performed on 2 ml of the solution and a Thinprep Pap test on the rest of the solution. Low-risk (LR)-HPV: 6, 11, 30, 40, 42, 43, 54, 61, 67, 73, 74, 81, 82, 83, 86, 87, 89, 90, 91; high-risk (HR)-HPV: 16, 18, 18v, 31, 33, 35, 35v, 39, 45, 51, 52, 56, 58, 59, and possibly high-risk (PHR)-HPV 26, 53, 66, 68a, 68b, 69, 70 were analysed with the Multiplex Luminex system (Bio-Rad Laboratories, Inc, Irvine, CA, USA) [1,2]. The HPV screening test was performed at the Laboratory of Infectious Epidemiology, Department of Laboratory Medicine, the Karolinska Institute, Stockholm, Sweden. Cytology was performed by biomedical technicians at the Department of Cytology and Pathology, the Sahlgrenska University hospital, Gothenburg, Sweden, and samples were prepared using the ThinPrep 5000 System (Hologic Inc) followed by Pap staining (Hologic). Samples classified as HSIL or cancer were re-evaluated by a pathologist. Cytology was reported according to the 2001 Bethesda System.

Sample preparation for proteomic analysis

Sodium dodecyl sulphate (SDS) solution (20%) was added to the samples in RNA-later until 2% final concentration, the samples were sonicated for 25 min and subsequently heated at 60°C for 5 min, followed by shaking on a FastPrep®-24 instrument (MP Biomedicals, OH, USA) for

5 repeated 40 seconds cycles at 6.5 m/s. Samples were centrifuged at 13 000 rpm for 10 min and the supernatants were transferred to clean tubes.

The samples were processed using the modified filter-aided sample preparation (FASP) method [3]. In short, the samples were transferred onto Nanosep 30k Omega filters (Pall Corporation, Port Washington, NY, USA) followed by centrifugation at 12 000 rpm for 10 min at 40°C to avoid the precipitation of salts and subsequently washed 3 times with 200 µl of 8 M urea at 40°C. The washed samples were reduced on filter with 100 mM DL-dithiothreitol (DTT) solution in 8 M urea for 1 h at 37°C, then washed twice with 200 µl of 8 M urea. Alkylation of the cysteine residues was performed using 10 mM methyl methanethiosulfonate (MMTS) solution in digestion buffer (0.5 % sodium deoxycholate (SDC), 50 mM triethylammonium bicarbonate (TEAB)) for 30 min at room temperature and the filters were then repeatedly washed with digestion buffer. Trypsin (Pierce Trypsin Protease, MS Grade, Thermo Fisher Scientific) in digestion buffer was added (300 ng per sample) and incubated at 37°C overnight; another aliquot of trypsin solution (300 ng) was added and the mixture was incubated at 37°C for 3 h. The peptides were collected by centrifugation and labelled using Tandem Mass Tag (TMTpro 16plex) reagents (Thermo Fischer Scientific) according to the manufacturer's instructions. The labelled samples were combined into the respective pools; the pooled samples were concentrated using vacuum centrifugation and SDC was removed by acidification with 10% TFA and subsequent centrifugation.

The combined TMT-labeled samples were fractionated into 40 primary fractions by basic reversed-phase chromatography (bRP-LC) using a Dionex Ultimate 3000 UPLC system (Thermo Fischer Scientific). Peptide separations were performed using a reversed-phase XBridge BEH C18 column (3.5 µm, 3.0 × 150 mm, Waters Corporation) and a linear gradient from 3% to 40% solvent B over 17 min followed by an increase to 100% B over 5 min. Solvent A was 10 mM ammonium formate buffer at pH 10.00 and solvent B was 90% acetonitrile, 10% 10 mM ammonium formate at pH 10.00. The primary fractions were concatenated into final 20 fractions (1 + 21, 2 + 22, … 20 + 40), evaporated and reconstituted in 15 µL of 3% acetonitrile, 0.2% formic acid for nanoflow LC-MS analysis.

LC-MS/MS Analysis

The fractions were analyzed on an Orbitrap Fusion Lumos Tribrid mass spectrometer interfaced with Easy-nLC 1200 liquid chromatography system (both Thermo Fisher Scientific). Peptides were trapped on an Acclaim Pepmap 100 C18 trap column (100 µm x 2 cm, particle size 5 µm, Thermo Fischer Scientific) and separated on an analytical column (75 µm x 35 cm, packed in-house with Reprosil-Pur C18, particle size 3 µm, Dr. Maisch, Ammerbuch, Germany) using a linear gradient from 5% to 33% B over 77 min followed by an increase to 100% B for 3 min, and 100% B for 10 min at a flow of 300 nL/min. Solvent A was 0.2% formic acid in water and solvent B was 80% acetonitrile, 0.2% formic acid. MS scans were performed at 120 000 resolution, *m/z* range 375–1500. The most abundant doubly or multiply charged precursors from the MS1 scans were isolated using the quadrupole with 0.7 *m/z* isolation window with a "top speed" cycle of 3 s and dynamic exclusion within 10 ppm during 45 seconds. The isolated precursors were fragmented by collision induced dissociation (CID) at 30% collision energy with the maximum injection time of 50 ms,

and detected in the ion trap, followed by multinotch (simultaneous) isolation of the top 10 MS2 fragment ions within *m*/*z* 400–1200, fragmentation by higher-energy collision dissociation (HCD) at 55% and detection of the MS^3 spectra in the Orbitrap at 50 000 resolution in the *m*/*z* range 100–500.

Proteomic Data Analysis

Identification and relative quantification were performed using Proteome Discoverer version 2.4 (Thermo Fisher Scientific). The database search was performed using the Mascot search engine v. 2.5.1 (Matrix Science, London, UK) against the Swiss-Prot *Homo sapiens* database (December 2019) with MS peptide tolerance of 5 ppm and fragment ion tolerance of 0.6 Da. Tryptic peptides were accepted with 1 missed cleavages. Methionine oxidation was set as a variable modification, cysteine methylthiolation, TMTpro on lysine and peptide N-termini were set as fixed modifications. Percolator was used for PSM and Protein ID validation with the strict false discovery rate (FDR) threshold of 1%.

Quantification was performed in Proteome Discoverer 2.4. TMTpro reporter ions were identified in the MS3 HCD spectra with 20 ppm mass tolerance, and the TMT reporter S/N values for each sample were normalized within Proteome Discoverer 2.4 on the total peptide amount (excluding the peptides from keratin and main plasma proteins). Only the unique identified peptides were taken into account for the relative quantification.

Statistics

All values from proteomics were logarithmized (base 2). When statistical significance was assessed between two groups, the Student´s paired t-test was used. When statistical significance was assessed between three groups, one-way analysis of variance (ANOVA) followed by the post-hoc Tukey HSD was used. To assess the diagnostic accuracy of biomarkers to identify high-risk lesions among low-risk and negative lesions or chronic HR-HPV lesions among cleared HR-HPV and negative lesions, receiver operating characteristic (ROC) curves were constructed where the area under the curve (AUC) with a 95% confidence interval (CI) was calculated. Here it was tested whether it was possible to distinguish a chronic HR-HPV from a cleared HR-HPV sample and negative sample combined or a high-grade lesion from a low-grade or negative sample combined. Binary logistic regression analysis was performed to assess the relationship between identified potential biomarkers and the dichotomous outcome chronic HR-HPV lesions vs. the combined group clearance of HR-HPV/negative lesions or high-grade lesion vs. low-grade/negative lesions. To find a biomarker panel with the highest sensitivity and specificity, the 2–4 biomarkers with the largest AUC were combined. Statistical significance was set at $p < 0.05$. IBM SPSS Statistics 25.0 (IBM Corp., Armonk, NY, USA) and GraphPad Prism program 8.4.3 (GraphPad Software, Inc., San Diego, USA) were used for statistical analyses.

Venn diagrams and volcano plots

Venn diagrams were created by using the interactive tool Venny 2.1 [4]. Volcano plots were created by plotting the negative log10 of the *p*-value against the log2 fold change between groups of comparison. To

identify statistically changed proteins with large magnitude changes, proteins with the longest distances from the origo of the volcano plot were identified (√ (x^2 + y^2). To identify the pre-defined seven potential biomarkers separating high-grade lesions from low-grade lesions and negative lesions on the volcano plots, the following calculations were performed:

$$\sqrt{[(-\log 10\ p\text{-value}_{\text{high-grade vs. neg}} - (-\log 10\ p\text{-value}_{\text{high-grade vs low-grade}}))^2 + (\log 2\ \text{fold-change}_{\text{high-grade vs. neg}} - \log 2\ \text{fold-change}_{\text{high-grade vs. low-grade}})^2]}$$

Supplemental figure and figure legends

All changed proteins

#term ID	term description	observed gene count	background gene count	strength	false discovery rate
GO:0043312	neutrophil degranulation	63	485	0.92	2.30×10^{-33}
GO:0016192	vesicle-mediated transport	106	1699	0.6	3.49×10^{-33}
GO:0002443	leukocyte mediated immunity	68	632	0.84	1.05×10^{-32}
GO:0002274	myeloid leukocyte activation	64	574	0.86	1.95×10^{-31}
GO:0002252	immune effector process	78	927	0.73	2.60×10^{-31}
GO:0002366	leukocyte activation involved in immune response	65	616	0.83	9.35×10^{-31}

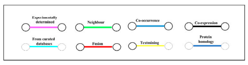

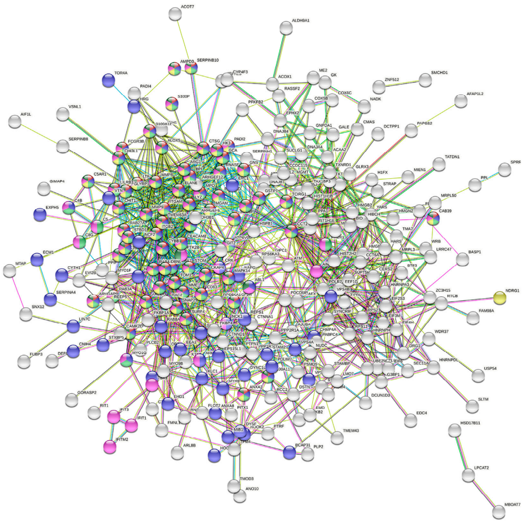

Figure S1. Protein-protein network analysis of the biological processes of all changed proteins in the incident HR-HPV groups. The biological processes or molecular processes with the lowest FDRs are indicated by colour and interactions are indicated by colour and line type. Immunoglobulin heavy constant gamma 2, Immunoglobulin heavy variable 1–46, Immunoglobulin heavy variable 1–18, Immunoglobulin heavy variable 1–24, Immunoglobulin heavy variable 3–64D, Immunoglobulin heavy variable 3–7, Immunoglobulin kappa variable 1–6, Immunoglobulin lambda variable 2–11, Immunoglobulin lambda variable 2–23, Immunoglobulin lambda variable 9–49 could not be identified in the String database. Disconnected nodes are hidden in the network.

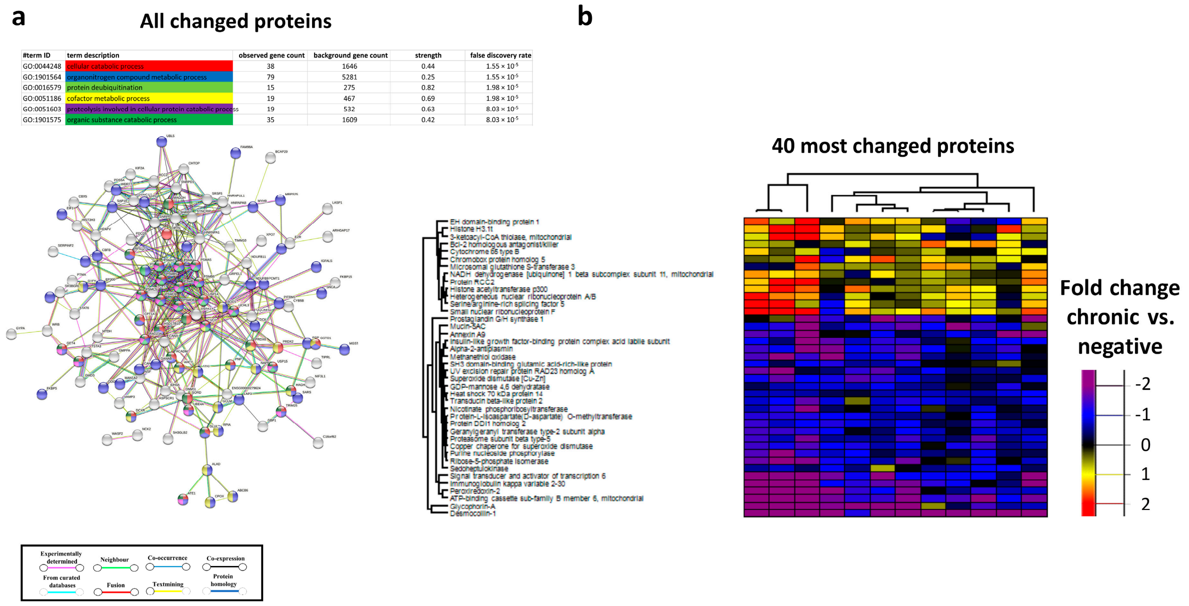

Figure S2. Protein-protein network analysis of the biological processes of all changed proteins in the chronic HR-HPV group and the HR-HPV negative group (**a**). The biological processes or molecular processes with the lowest FDRs are indicated by colour and interactions are indicated by colour and line type; Heatmap of the 40 most changed proteins between the chronic HR-HPV group *vs.* the HR-HPV negative group (**b**). Immunoglobulin kappa variable 2–30 could not be identified in the String database. Disconnected nodes are hidden in the network.

References

1. Munoz, N.; Bosch, F.X.; de Sanjose, S.; Herrero, R.; Castellsague, X.; Shah, K.V.; Snijders, P.J.; Meijer, C.J.; International Agency for Research on Cancer Multicenter Cervical Cancer Study, G. Epidemiologic classification of human papillomavirus types associated with cervical cancer. *N Engl J Med* **2003**, *348*, 518-527, doi:10.1056/NEJMoa021641.
2. Schmitt, M.; Dondog, B.; Waterboer, T.; Pawlita, M. Homogeneous amplification of genital human alpha papillomaviruses by PCR using novel broad-spectrum GP5+ and GP6+ primers. *J Clin Microbiol* **2008**, *46*, 1050-1059, doi:10.1128/JCM.02227-07.
3. Wisniewski, J.R.; Zougman, A.; Nagaraj, N.; Mann, M. Universal sample preparation method for proteome analysis. *Nat Methods* **2009**, *6*, 359-362, doi:10.1038/nmeth.1322.
4. Oliveros, J.C. An interactive tool for comparing lists with Venn's diagrams; Available online: (https://bioinfogp.cnb.csic.es/tools/venny/index.html. Accessed on 26 November 2021)
